# Sleep and temperature data from wearable devices support noninvasive detection of diabetes mellitus in a large-scale, retrospective analysis

**DOI:** 10.1038/s43856-026-01501-0

**Published:** 2026-03-16

**Authors:** Varun K. Viswanath, Shreenithi Navaneethan, Jamison H. Burks, Severine Soltani, Patrick Kasl, Wendy Hartogensis, Stephan Dilchert, Frederick M. Hecht, Ashley E. Mason, Edward J. Wang, Benjamin L. Smarr

**Affiliations:** 1 Department of Electrical and Computer Engineering, Jacobs School of Engineering, University of California, San Diego, CA USA; 2 Shu Chien – Gene Lay Dept. of Bioengineering, Jacobs School of Engineering, University of California, San Diego, CA USA; 3 Osher Center for Integrative Health, University of California, San Francisco, CA USA; 4 Department of Management, Zicklin School of Business, Baruch College, The City University of New York, New York, NY USA; 5 Halıcıoğlu Data Science Institute, University of California, San Diego, CA USA

**Keywords:** Predictive markers, Diagnosis

## Abstract

**Background:**

Diabetes Mellitus is a common, chronic metabolic disorder affecting the cardiovascular system, autonomic nervous system, and sleep quality. Diabetes affects diverse physiological data including heart rate variability, distal body temperature, and sleep duration. We hypothesized that biologically informed features from wearable device data, combined with appropriate application of longitudinal data, can capture physiological covariates of diabetes and support the noninvasive detection of diabetes.

**Methods:**

We obtained 4 months and 7 days of wearables data (Oura Ring) from 389 individuals self-reporting diabetes and 10,820 people self-reporting no diabetes diagnosis from the TemPredict database. We selected 36 features of sleep, circadian disruption, and distal body temperature from literature and evaluated whether time windows of these features could be classified to be from individuals self-reporting diabetes (N = 236) or self-reporting no diabetes diagnosis (N = 282).

**Results:**

Here we show longer time windows of input perform better, with the best algorithm (21-nights) achieving 0.88 Area under ROC (AUROC) and 0.80 Area under Precision Recall (AUPRC) (0.30 improvement over random). Feature analyses reveal the importance of further derived distal body temperature features (increase AUROC by 0.0724), especially to differentiate other chronic conditions from diabetes. The model achieves 0.80 AUROC and 0.28 improvement over random in AUPRC in an imbalanced cohort drawn from 6,658 individuals, emulating a general population.

**Conclusions:**

These results indicate the value of biologically informed features and longitudinal data for identifying people with diabetes and further, suggest that these methods could make such separations possible for other chronic conditions that affect sleep and inflammation.

## Plain Language Summary

Diabetes mellitus is a condition where the body cannot properly regulate blood sugar. It affects multiple organ systems. We evaluated whether these effects can be seen in data collected from noninvasive wearable devices (e.g., Oura Ring, Apple Watch). We processed wearables data from 11,209 individuals. We then evaluated whether this data could be used to identify individuals that have diabetes. We tested multiple computational models that separate data from people with diabetes from data from people with no diabetes diagnosis. Incorporating knowledge about their sleep, physiology and 21 nights of device data improved identification of people with diabetes. This shows the feasibility of detecting physiology consistent with diabetes from real-world, noninvasive wearables data, which could be helpful to diagnose and monitor people with diabetes.

## Abstract (web summary)

First author et al. study the physiological effects of diabetes mellitus in data collected from noninvasive wearable devices (e.g., Oura Ring, Apple Watch) and test whether machine learning can identify these effects. Biologically informed features (e.g., sleep and temperature) improve AUROC from 0.808 to 0.880

## Introduction

Diabetes mellitus (DM) is a chronic metabolic disorder with a complex pathogenesis^1^. DM is a metabolic disorder that is characterized by dysregulated blood glucose levels (i.e., hyperglycemia and hypoglycemia), which in turn can lead to harmful physiological effects in the gastrointestinal, renal, nervous, and cardiovascular systems. Common tests to identify DM and prediabetes include HbA1c, random blood glucose, and fasting blood glucose measurements. Although these tests can be used to assist health professionals in confirming a DM diagnosis, they require that either the professional or the patient themselves have noticed DM symptoms in the first place. Of the estimated 37.1 million adults with DM in the United States, roughly 8.5 million (23%) are undiagnosed^1^. For the estimated 96.0 million adults with prediabetes, only about 17.4% are aware that they have the condition^1^. Further, while continuous glucose monitoring (CGM) devices are reliable tools for DM management, many undiagnosed individuals do not seek out even a minimally invasive device without a specific diagnosis. There is a clear need for more accessible tools that could aid public health professionals to predict who is developing DM or prediabetes so that potential patients can seek proper diagnosis and treatment.

A new wave of widely adopted wearable health devices (wearables) could noninvasively measure the physiological and behavioral effects associated with DM and prediabetes. Both DM and prediabetes are independently associated with lower heart rate variability (HRV), which suggests that cardiac autonomic dysfunction may be characteristic of prediabetes^2–5^. Furthermore, people with DM have been reported to have lower skin blood flow and dysregulated thermoregulatory responses during heat exposure, which is associated with dysregulated thermal, cardiovascular, and glycemic control^6–8^. Decreased blood flow to the skin and decreased peripheral uptake of glucose lead to reductions in skin temperature in response to environmental heat or food intake. DM is also intimately related to chronic inflammation^9^, which can lead to noticeable increases in body temperature, especially as a pattern of change across time. Beyond physiological measurements that are directly controlled by the autonomic nervous system, DM can affect behaviors. A bidirectional relationship exists between DM risk and sleep quality: chronic sleep disturbances can elevate the risk of developing insulin resistance, while DM can worsen the quality of sleep^10^. These systemic physiological effects of DM and prediabetes could be used as indicators of diabetic propensity. Wearables measure heart rate (HR), HRV, and breathing rate through photo-plethysmography (PPG) sensors and distal body temperature through temperature sensors. Appropriate featurization of these physiological data over time could enable wearables to capture the chronic effects of DM and become a continuous and noninvasive tool for DM detection and possibly longitudinal monitoring^11^.

Early studies that used PPG to identify people with DM focused on characteristics of the PPG waveform^12–19^. Later work shifted towards using smartphones or specialized devices to measure PPG signals and machine learning approaches to identify people with DM, hyperglycemia, or early detection of hypoglycemia^20–25^. These works developed approaches from at most 100 individuals and in most cases, recorded less than a day of data. One work used a large cohort of 2500 subjects, but only recorded 2 min of data per individual. More recently, wearables (e.g., Empatica E4 Smartwatch) have enabled longitudinal data collection over multiple days. Features of HR, HRV, movement, and electrodermal activity (EDA; a measure of perspiration) extracted from wearable data have been applied to detect hypoglycemic events in cohorts ranging from 1 to 36 individuals^5,26–28^. Skin temperature was additionally collected from wrist-worn wearables was assessed for changes with hyperglycemic events in cohorts ranging from 10 to 20 individuals^29–32^. Temperature measured on the skin of the extremities (hands or feet), which is typical of commercially available wearables, is defined as distal body temperature^33^. These works analyzed how changes in statistical measures of distal body temperature can be used for identifying hypoglycemic events and prediction blood glucose concentration^29–32^.

Many gaps exist in the prior work. First, the combination of features from multiple data streams is essential because it holds the potential for improved separation between physiology consistent with DM and other states. Second, statistical measures of distal body temperature feature poorly capture information carried in the diurnal and ultradian timescales of this data stream. Distal body temperature is a physiological function of core body temperature, vascularization in the extremities, coupled with sleep cycles. Diurnal features of distal and core body temperature have been central to analyses of circadian rhythm^33,34^; studied as measures for fever, depression, muscular work^33,35–37^; and revealed to be associated with future disease risk for a breadth of conditions (including Type 2 Diabetes) in a study of the UKBioBank^38^. Diurnal amplitude in distal body temperature measured by an Oura Ring was associated with depression^39^, possibly due to its sensitivity to thermoregulatory dysregulation. It evidently captures marked physiological effects of DM. Within a night, the complexity of the distal body temperature time series has been related to the autonomic nervous system’s (ANS) health during sleep^6,34^. Multiscale analyses and features of entropy elucidate more information related to the complexity of a data stream than statistics alone^40–42^. Similarly, features of sleep-wake time series can directly capture insomnia-like patterns in sleep related to DM^6,34,43,44^. Finally, all prior works either used short recordings (usually only a couple minutes) from a single day as input or evaluated their methods with small cohorts (23–100 subjects), raising the question of whether these methods maintain their performance over time (over weekly and monthly timescales) and across individuals (as the effects of DM can differ with ages and comorbidities).

This work analyzed 4 months and 7 days of data from 389 people who self-reported DM and 10,820 people who self-reported having never received a DM diagnosis (DX) from a healthcare professional and, therefore, is the largest-scale, longitudinal analysis of DM detection using noninvasive physiological data from wearable devices to date. We assessed whether people who self-reported DM manifest differences in physiological data recorded with an Oura Ring, which noninvasively tracks several minute-to-minute physiological “data streams”: HR, HRV, sleep, and distal body temperature (measured on the skin of the finger). Additionally, while previous work focused on HR and HRV^22–25^, we extracted diurnal and ultradian features of distal body temperature and sleep and assessed whether these features reveal complementary information to HR and HRV about physiology consistent with DM^31^.

We first tested for population differences in each data stream with statistical methods. We then tested whether a machine learning algorithm using biologically informed features of each data stream could detect when multiple-night time windows were drawn from an individual who self-reported DM. We optimized the input window size and model characteristics with a balanced cohort of 282 individuals self-reporting no DM DX and 236 individuals self-reporting DM, and then analyzed feature importance as well as overall performance. Finally, we demonstrated the performance of the model on an imbalanced dataset of 87 reporting DM DX and 6571 reporting no DM in order to emulate a general population. These analyses demonstrate that non-invasive methods to detect DM can be effective and robust with repeated measures over time and biologically informed features skin temperature and sleep.

## Methods

### Data collection

Our data were collected through the TemPredict study at the University of California, San Francisco, in collaboration with the University of California, San Diego. The University of California, San Francisco (UCSF) Institutional Review Board (IRB, IRB# 20-30408) and the U.S. Department of Defense (DOD) Human Research Protections Office (HRPO, HRPO# E01877.1a) approved of all study activities, and all research was performed in accordance with relevant guidelines and regulations and the Declaration of Helsinki. All participants provided informed consent (electronic). We did not compensate participants for participation.

We collected Oura Ring Gen2 data from 63,153 participants from January to December of 2020. Further details are contained in Mason et al.^21^. A baseline self-report survey collected comorbidity information, including whether or not the individual had ever been diagnosed as a person with DM. Participants could respond yes, no, or refrain from responding. Sleep stages (4-stage; light NREM sleep, deep NREM sleep, REM sleep, and wake) at 30-s granularity and sleep/wake onset (start and end times of a series of contiguous sleep stage predictions) were obtained for each night of sleep from the Oura Ring. The Oura Ring predicts sleep stages with a proprietary algorithm that has been validated against Polysomnography (PSG) and Electro-encephalogram (EEG) by Miller et al., Ghorbani et al., and de Zambotti et al. ^45–47^. The Oura Ring noninvasively tracks 4 minute-to-minute physiological “data streams”: HR, HRV (typically calculated from a sliding 5-min window), sleep, and distal body temperature (measured on the skin of the finger). The HR and HRV data have been validated by prior work^48^. Data for all physiological and sleep stage data streams were available for 33,374 individuals, of which 389 self-reported having DM, and 10,820 self-reported having never received a DM diagnosis (DX) from a healthcare provider. *N* = 21,402 individuals did not report whether or not they have DM, and nights from these individuals were not used in the analysis. Survey data were missing for 763 individuals.

**Fig. 1:**
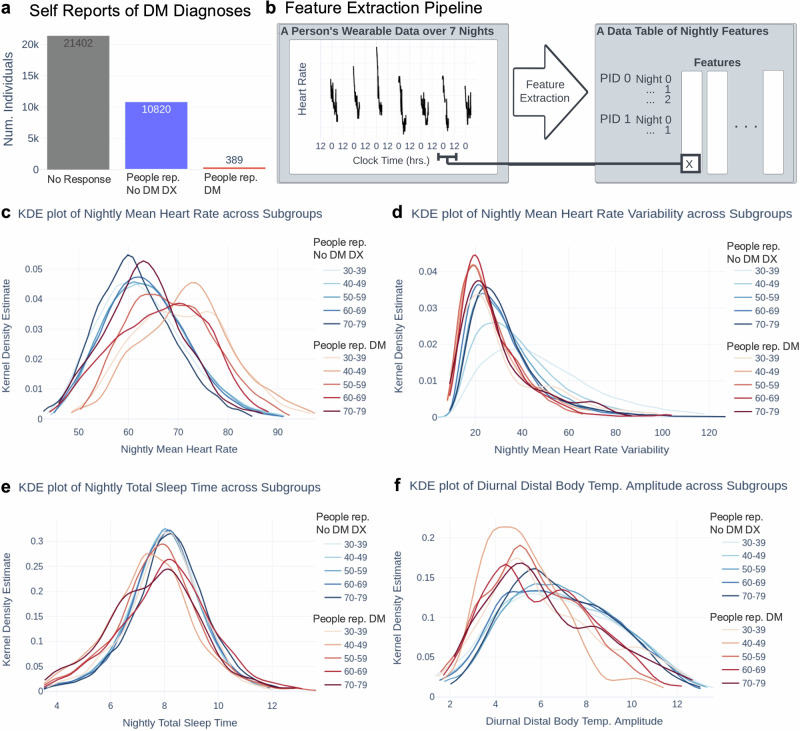
Population level comparisons between people self-reporting DM and people self-reporting no DM DX. **a** Number of people self-reporting DM, people self-reporting no DM DX, and No Response reports in our dataset. **b** Feature extraction of nightly features. The left panel shows one week of nightly measurements during sleep, with sleep intervals typically spanning midnight (time 0) and ending before noon (time 12). **c**–**f** Kernel density estimates of the commonly used features (hr_average, rmssd, total_sleep_time, diurnal_distal_body_temperature_amplitude) by each age group for both populations reporting DM DX status. “People self-reporting DM”: red; “People self-reporting no DM DX”: blue. Darker/more saturated lines indicate older age; lighter lines indicate younger age.

**Fig. 2:**
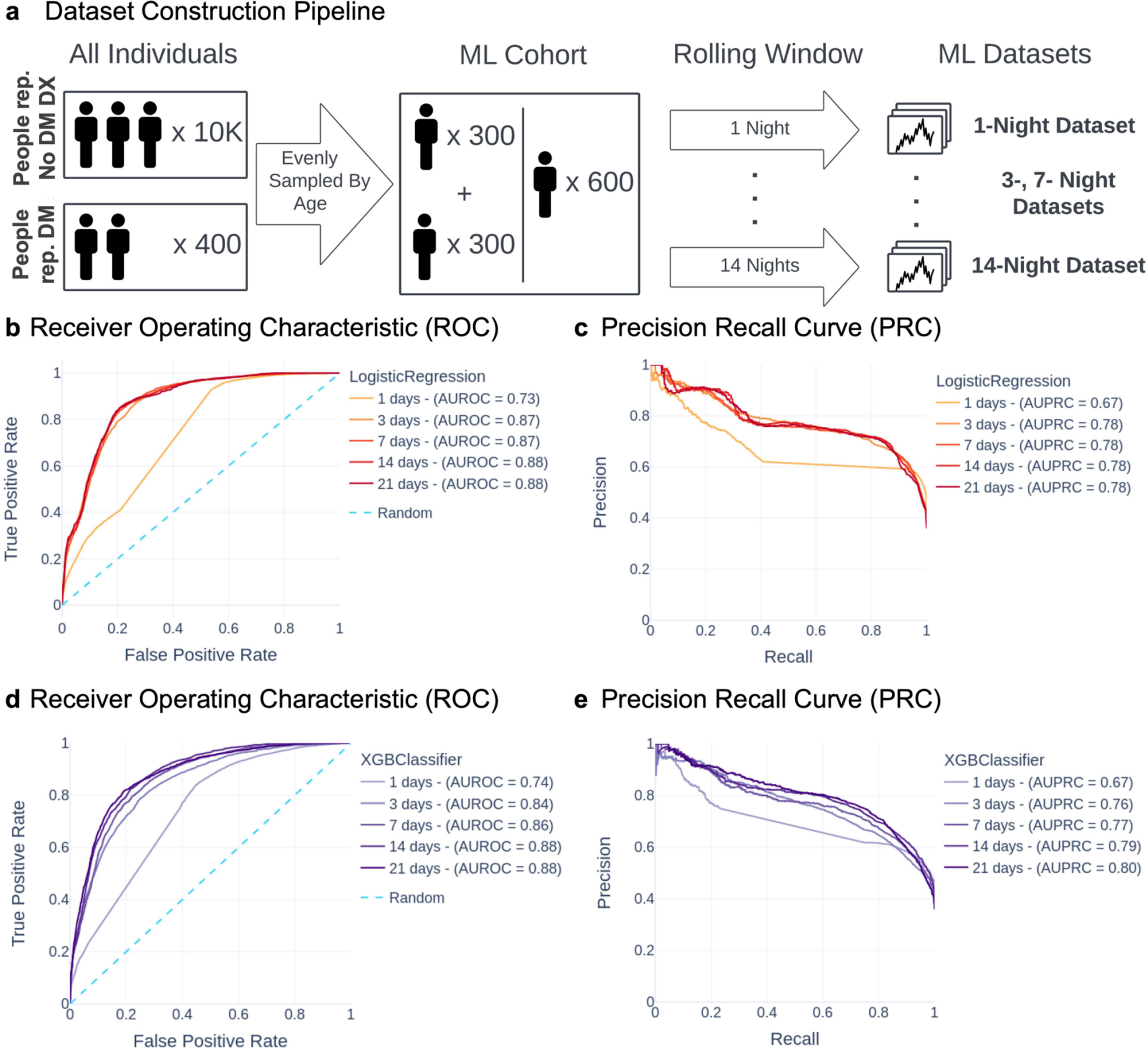
Machine learning algorithms classify time windows of physiological data from people self-reporting DM. **a** Construction of machine learning datasets from cohorts of people self-reporting DM and people self-reporting no DM DX. Datasets vary in the length of input time windows. **b**–**e** ROC curves and PRC for each ML model used, over a range of different input time windows. Longer time windows are shown in darker shades. Gradient boosting classifier results are shown in purple. Logistic regression classifier results are shown in orange.

**Fig. 3:**
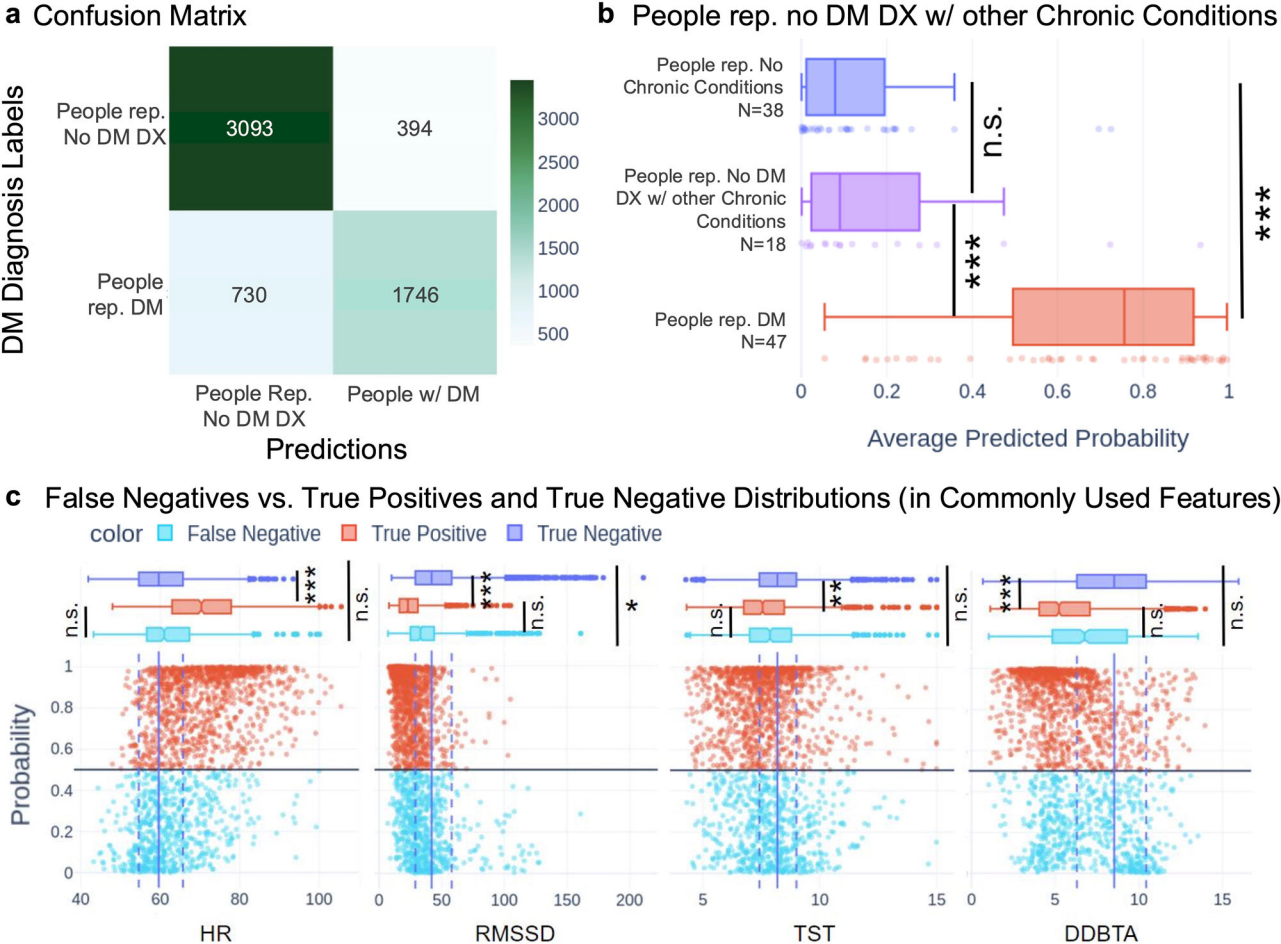
Analysis of false positives and confounding variables in predictions from the best machine learning algorithm. **a** Confusion matrix for best performing model (21-night XGBClassifier). Darkness is proportional to the number of time windows in each category. **b** Average probability of a positive prediction in people self-reporting DM, people self-reporting no DM DX with other chronic conditions, and people self-reporting no chronic conditions. Red vs. Purple: *p* = 7.0 e-6; red vs. blue: *p* = 6.9 e-12 (see Supplementary Table 1). **c** Distributions of false negatives and true positives, shown in comparison to the true negative distribution. Exact *p*-values in Supplementary Table 2. All statistical test results shown in this figure were generated from Dunn’s Test, using Benjamini–Hochberg *p*-value threshold correction. * indicates a *p*-value < 0.05, ** indicates a *p*-value < 0.01, *** indicates a *p*-value < 0.001, and n.s. (not significant) indicates a *p*-value ≥ 0.05.

**Fig. 4:**
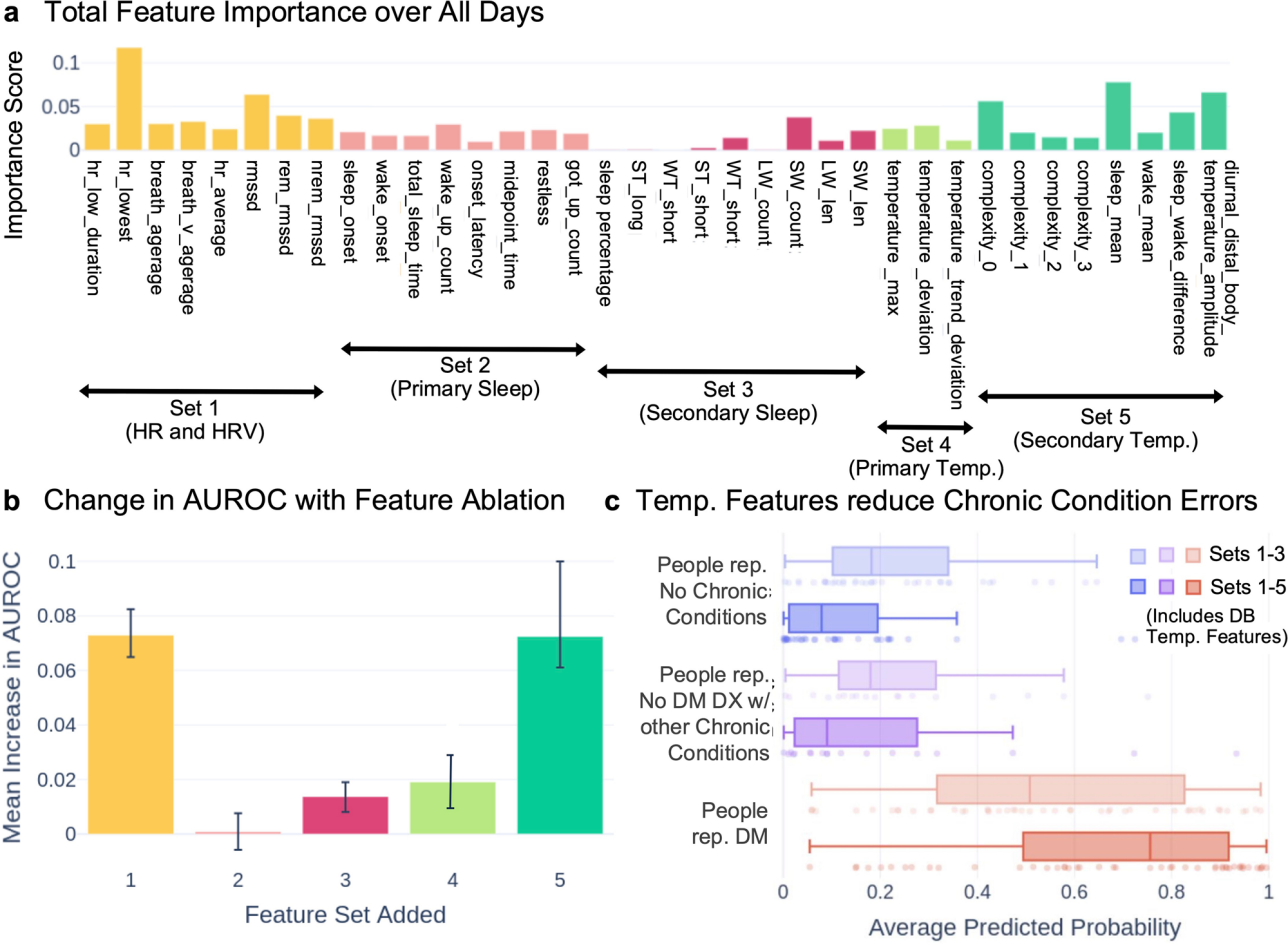
Feature importance and ablation analyses of best machine learning algorithm. **a** Feature weights for each feature averaged across all the nights in the input window for the best performing model. **b** Mean improvement in classifier performance as a result of adding each feature set. Error bars represent 25th and 75th quantiles of the increase in AUROC upon addition of each set (*n* = 16 possible feature set additions per feature). **c** Reduction in classification errors in people self-reporting no DM DX with other chronic conditions, as a result of adding feature sets 4 and 5.

We extracted a set of features for each night using minute-level physiological data between sleep onset and wake onset. These features are described in detail in the next section. *N* = 36,061 nights from people self-reporting DM and 1,086,096 nights from people self-reporting no DM DX were considered. Nights where we were unable to extract the complete set of our features (e.g., device out of battery, too many missing values to compute any feature) were dropped. In addition, age bins 18–19, 20–29, and 80+ were dropped due to low sample sizes that would not allow for the determination of significant differences in those age groups. Data filtering after feature extraction resulted in 24,486 nights from 315 people self-reporting DM and 764,315 nights from 9063 people self-reporting no DM DX with 36 features. This resulted in 84.3 nights per individual (65.3% data completeness), with 84.6 nights per individual (65.6% data completeness) from individuals who self-reported no DM DX, and 77.7 nights from individuals (60.2% data completeness) who self-reported DM. Data completeness was calculated as a fraction of nights data was collected for (129 nights). Age and sex data are reported in Table 1. Additional ethnicity data is reported in Supplementary Table 7, though this variable was not used in our analyses because our population was majority white (83.55%). We presented our initial dataset statistics in Fig. 1 with these nightly features. The machine learning experiments from Figs. 2, 3, and 4 were performed with a stratified subsample of this dataset that we describe in the *Machine Learning* subsection of our methods.

**Table 1:** Demographic data by label and granularity

		Num. Individuals			Num. Nights from Individuals		
Age Bin	Sex	Rep. No DM DX	Rep. DM	Percent rep. DM (%)	Rep. No DM DX	Rep. DM	Percent rep. DM (%)
**30–39**	**M**	1322	27	2.0	110,063	1624	1.5
	**F**	694	13	1.8	58,723	977	1.6
	**O**	2	0	0.0	137	0	0.0
	**T**	**2018**	**40**	**1.9**	**168,923**	**2601**	**1.5**
**40–49**	**M**	1546	61	3.8	129,250	5193	3.9
	**F**	994	20	2.0	84,322	1683	2.0
	**O**	1	0	0.0	119	0	0.0
	**T**	**2541**	**81**	**3.1**	**213,691**	**6876**	**3.1**
**50–59**	**M**	1076	71	6.2	94,210	5409	5.4
	**F**	843	24	2.8	73,814	1980	2.6
	**O**	2	0	0.0	65	0	0.0
	**T**	**1921**	**95**	**4.7**	**168,089**	**7389**	**4.2**
**60–69**	**M**	500	45	8.3	44,964	3573	7.4
	**F**	406	22	5.1	37,833	1833	4.6
	**O**	1	0	0.0	78	0	0.0
	**T**	**907**	**67**	**6.9**	**82,875**	**5406**	**6.1**
**70–79**	**M**	183	20	9.9	15,829	1299	7.6
	**F**	139	5	3.5	12,740	462	3.5
	**O**	0	0	-	0	0	-
	**T**	**322**	**25**	**7.2**	**28,569**	**1761**	**5.8**
**Total**	**M**	**4627**	**224**	**4.6**	**394,316**	**17,098**	**4.2**
	**F**	**3076**	**84**	**2.7**	**267,432**	**6935**	**2.5**
	**O**	**6**	**0**	**0**	**399**	**0**	**0**
	**T**	**9063**	**315**	**3.5**	**764,315**	**24,486**	**3.2**

Sample size for each age group in statistical testing of individuals features for differences between individuals self-reporting no DM DX and individuals self-reporting DM. (M Male, F Female, O Other, and T Total). The totals for each sex across all age groups are reported in the last 4 rows labeled “totals”. Rep. DM = people who self-reported diabetes mellitus, Rep. no DM DX = people self-reporting no diagnosis of diabetes mellitus. The subtotal rows are bolded and subtotal values for “Percent Rep. DM” columns are the percentages based on the subtotal counts for that row.

### Feature extraction

We extracted features (mathematical functions that extract relevant information) from the minute-level physiological data streams of each night (using data between sleep onset and wake onset based on the Oura Ring sleep stage algorithm predictions). We group features as shown in Table 2. Feature Sets 1, 2, and 4 were generated by a proprietary algorithm to which we do not have access. Feature Sets 3 and 5 were extracted from minute-level data streams following methods described in prior work. We refer to the features from prior work as “secondary” and the proprietary features as “primary” because the secondary features are further derived from the data stream that they were extracted from than the primary features. The secondary features typically also require more calculations than the primary features.

**Table 2:** Groupings of features used in feature importance analyses

Feature sets									
HR and HRV (Set 1)		Primary sleep (Set 2)		Secondary sleep (Set 3)		Primary temp. (Set 4)		Secondary temp. (Set 5)	
1	hr_low_duration	9	sleep_onset	17	sleep_percentage	26	temperature_max	29	sleep_mean
2	hr_lowest	10	wake_onset	18	ST_long	27	temperature_deviation	30	wake_mean
3	breath_average	11	total_sleep_time	19	WT_long	28	temperature_trend_deviation	31	sleep_wake_difference
4	breath_v_average	12	wake_up_count	20	ST_short			32	diurnal_distal_body_temp_amp
5	hr_average	13	onset_latency	21	WT_short			33	complexity_0
6	rmssd	14	midpoint_time	22	LW_count			34	complexity_1
7	rem_rmssd	15	restless	23	SW_count			35	complexity_2
8	nrem_rmssd	16	got_up_count	24	LW_len			36	complexity_3
				25	SW_len				

All extracted features were grouped based on the minute-level physiological time series they were extracted from and the level of derivation of the feature. These groupings were used to enhance the interpretability of our feature importance and ablation analyses. More information on each feature is in our Supplementary Data.

We identified four “commonly used features” that are tied with each measured data stream: average resting HR (beats per minute; BPM), average resting HRV (root mean square of successive differences; RMSSD), total sleep time (TST) in hours, and diurnal distal body temperature amplitude (DDBTA) in degrees Celsius. We refer to these as the “commonly used features” because they are features of a data stream commonly used as indicators for a variety of remote health monitoring and wellness settings that capture changes in the data stream relevant to DM at the nightly level^31–33,35–38,45,49^. Then, we compared the nightly commonly used feature values of the population self-reporting DM with those of the population self-reporting no DM DX.

#### Oura summary features (Sets 1, 2, and 4)

Oura constructed these features with a proprietary algorithm. These features are available through their public API. We divided these features into three groups based on their association with a particular aspect of physiology. Feature set 1 consisted of features associated with HR and HRV (Supplementary Data 1). Feature set 2 consisted of primary features associated with characteristics of sleep behavior (Supplementary Data 1). Feature set 4 consisted of features associated with distal body temperature (Supplementary Data 1). Feature sets 1, 2, and 4 were included as they might be indicative of autonomic dysfunction, disruptions in thermoregulatory function, and chronic sleep disturbances, respectively, as previously mentioned, and might capture diabetic propensity.

HR and HRV features were calculated from sliding windows, as indicated in Supplementary Data 1, and have been validated by prior work^48^. Sleep onset, wake onset, and total sleep time were extracted from the predicted timestamp for wake onset and sleep onset. These features were calculated as hour + minute/60, and total sleep time is the circular difference of wake onset and sleep onset.

#### Secondary sleep features (Set 3)

The secondary sleep features (Supplementary Data 2) were extracted based on prior work by Katori et al. and Viswanath et al.^43,44^ that aimed to develop sleep phenotypes (digital phenotypes focused on sleep) from nightly activity data that better diversified the variety of sleep behaviors people exhibit. We used hypnograms containing 30-s predictions of sleep vs. wake throughout the night. These data were obtained directly from Oura Health Oy. We then followed the steps outlined by Katori et al. and Viswanath et al. to extract sleep features. We first identified long windows (>6 h) and short windows (<3 h) of contiguous sleep (no wake periods longer than 10 min) within each night. Windows were separated by at least 60 min of wake time. Then, features for each night were extracted, representing the number of long and short windows (LW_count, SW_count), the time in hours of sleep time and wake time from long windows (ST_long, WT_long) and short windows (ST_short, WT_short), and the time in hours of long and short windows (LW_len, SW_len). Sleep efficiency was calculated as total sleep time divided by total wake time (sleep_percentage). The length, sleep time, and wake time features were coded as missing if the type of window they were associated with did not occur in a night. Katori et al. showed that these features better represent sleep behaviors associated with insomnia-like sleep, and Viswanath et al. further associated these features with DM, sleep apnea, and respiratory illness. Thus, it was likely that they could better identify individuals self-reporting DM than the Oura Ring sleep features alone.

#### Secondary temperature features (Set 5)

The secondary temperature features (Supplementary Data 3) consist of two approaches to extracting features from minute-level distal body temperature data. The first approach focused on differences between daytime and nighttime distal body temperature, aiming to capture circadian rhythm instability. Since circadian rhythmicity is affected by DM, we aimed to test the applicability of these temperature features in this different context. The second approach draws on advances in information theory and distance metrics that aim to characterize the complexity of a signal^50^. The complexity of the distal body temperature time series has been related to the health of a person’s ANS during sleep^6,34^, and we hypothesized this association could be observed using temperature data from a finger-worn wearable device. Thus, we tested whether multiscale complexity is useful in detecting DM^42^.

The first approach aimed to characterize circadian rhythm stability. We used predicted sleep and wake onset data (described as part of Set 2) to determine periods when a person was asleep versus awake. As done in a prior study of body temperature and depression^39^, we computed metrics for the daily average distal body temperature during minutes awake (wake_mean), the daily average during sleeping minutes (sleep_mean), and the difference between those averages (sleep_wake_difference). To compute the DDBTA (diurnal_distal_body_temp_amp), we calculated the daily maximum distal body temperature as the highest daily value and the daily minimum distal body temperature as the lowest daily value for each person and calculated the difference between these two values.

For each 24-h period, we discarded wearable sensor-assessed distal body temperature values below the 5th percentile value and above the 95th percentile. This served to exclude periods of non-wear and periods of clinical fever and to minimize influence from non-representative extreme values (e.g., outliers), as done in prior research with wearable sensor-assessed metrics^51,52^.

The second approach aimed to characterize the complexity of the sleep time distal body temperature time series^42^. Complexity is related to the underlying structure of the time series and its information content. It has been shown that measures of complexity in HRV are related to differences across ages as well as between healthy and disease states. Because DM affects physiological control mechanisms related to nocturnal thermoregulation, we hypothesized that the Complexity Index (CI) might reveal differences between individuals self-reporting DM and individuals self-reporting no DM DX. Complexity index is a measure derived from multiscale analysis, an area which seeks to uncover information about the underlying structure of time series data. This measure is used here to extract information about the dynamics of the distal body temperature data stream during sleep at varying timescales. The complexity features (complexity_0, complexity_1, complexity_2, complexity_3) were extracted based on the CI method presented by Batista et al.^40^ and the multi-scale coarse-graining method presented by Costa et al.^41^; the combination of which is described by Burks et al.^53^. We describe these two methods first to facilitate our description of our own methods. The calculation of the CI was derived from work established by Batista et al. In summary, the metric is used to determine the “effective” length of a time-series if it were to be extended until flat. More formally, for a discrete, uniformly sampled time-series X(t) where t ranges from 0 to T, the CI can be calculated as:

$$C{I}_{X\left(t\right)}=\sqrt{{\sum }_{{{{\rm{t}}}}=0}^{{{{\rm{T}}}}-1}{\left({{{{\rm{x}}}}}_{{{{\rm{t}}}}+1}-{{{{\rm{x}}}}}_{{{{\rm{t}}}}}\right)}^{2}}$$

To ensure that the comparison of CI is consistent across time-series with substantially differing variances, all data is z-scored, and the CI is normalized by the quantity of samples used in its calculation

Multi-scale coarse graining is the process of producing several new time-series by reducing the length of an original time-series by using an aggregation function (we use the mean) over a subset of samples, w, based on a varying window size, W. Typically, window sizes are multiples of 2 to ensure no data points are unnecessarily dropped at different integer scales, s. More formally, for each scale in a time-series of size N, the coarsely grained values of that scale can be calculated as:

$$\widetilde{{{{{\rm{g}}}}}_{{{{\rm{i}}}}}^{{{{\rm{s}}}}}}=\frac{1}{{{{{\rm{W}}}}}_{{{{\rm{s}}}}}}{\sum }_{{{{\rm{j}}}}=0}^{{{{{\rm{W}}}}}_{{{{\rm{s}}}}}-1}{X}_{\left(i\cdot {W}_{s}\right)+j}{\mbox{where}}{{{{\rm{W}}}}}_{{{{\rm{s}}}}}={2}^{{{{\rm{s}}}}}$$

$$\widetilde{{G}_{{{{\rm{s}}}}}}=\{\widetilde{{{{{\rm{g}}}}}_{1}^{{{{\rm{s}}}}}},\widetilde{{{{{\rm{g}}}}}_{2}^{{{{\rm{s}}}}}},..,\widetilde{{{{{\rm{g}}}}}_{{{{\rm{N}}}}/{{{{\rm{W}}}}}_{{{{\rm{s}}}}}}^{{{{\rm{s}}}}}}\}$$

to create a new, coarsely grained time-series, G. The multiple coarsely grained time series represent the physiologic time series at multiple time scales. When we calculate the CI on the new, coarsely grained time-series, we obtain the multi-scale complexity indices.

In this work, we calculated the CI at the 0th, 1st, 2nd, and 3rd scales (with the 0th scale being the original time series, the 1st scale being the coarse-grained signal of every two samples, and so on) from the distal body temperature time series of each night (complexity_0, complexity_1, complexity_2, complexity_3)^53^. Our aim was to assess whether the impact of DM on the ANS, which affects the nocturnal thermoregulation, could be observed as a change in the underlying structure of the distal body temperature signal. We selected 256 samples around the midpoint of the night (determined by sleep_onset and wake_onset). We assessed a little over 4 h from the center of the night to avoid variance around wake and sleep onset that could bias our assessment of the time series’ underlying structure. We performed multiscale coarse-graining using the mean as the aggregation function from non-overlapping windows and calculated CI from the 0th, 1st, 2nd, and 3rd grains (interchangeable term for scales). Each complexity feature represents how complex the signal is at a certain temporal resolution. Complexity_0 represents the complexity at the minute-level resolution, while complexity_3 represents a 16-min resolution. At each resolution, the feature approximates how much information might be in the signal based on point-to-point jaggedness. For more details, see Costa et al.^40^, Batista et al.^41^, and Burks et al.^53^.

#### Statistical testing for differences in individual features

We tested whether each extracted feature differs in individuals who self-reported DM from individuals who self-reported no DM DX using the Mann–Whitney U test for difference in distribution, 2-sided. As stated previously, these tests were performed with 764,315 nights from people self-reporting no DM DX and 24,486 nights from people self-reporting DM. Sample sizes for statistical testing within age groups are shown in Table 1.

The commonly used features were preprocessed before we calculated the kernel density estimates for Fig. 1c–f. HR mean was filtered outside of the 5 and 95% quantiles. HRV mean was filtered outside of the 98% quantile. Total sleep time was filtered outside of the 1 and 99% quantiles. 24-h circadian amplitude was not filtered. All features had missing values dropped after filtering.

### Machine learning

#### Preprocessing and data quality control

We tested whether machine learning algorithms could detect DM using combinations of our nightly features in short time windows. We first constructed a controlled dataset. We controlled for time of year by limiting data extraction to data falling within a specified date range: 2020-04-22 to 2020-08-29. We chose these dates because we have data for at least 50% of individuals on every night between these dates (see *Methods > Data Collection*), and because it limits variability due to seasonal changes (contained to late spring and summer). We then performed stratified sampling based on age and sex to obtain a balanced number of individuals self-reporting DM and individuals self-reporting no DM DX in each stratum, for a total of 300 individuals self-reporting DM and 300 individuals self-reporting no DM DX.

Because some of our experiments would include data quality standards that would result in further reductions in the number of samples in our dataset, we preemptively applied the most strict of these standards on our data so that they would affect all our experiments. Specifically, we planned to experiment with input time windows of differing lengths. Across all possible 21-night time windows, we dropped windows with more than 2 missing nights in any week. We used the start dates of the 21-night time windows that passed this criteria to construct the datasets with time windows of other lengths. After applying this filtering, we obtained our machine learning dataset consisting of 236 individuals self-reporting DM with 10,621 nights and 282 individuals self-reporting no DM DX with 21,104 nights, with 36 features extracted for each night. A scaler was fit to the training data (with missing values excluded) and used to transform the validation and testing data. All missing values were then zero-filled. We trained statistical learning models that combined the nightly features values from a short time window to predict whether the window was extracted from an individual self-reporting DM or an individual self-reporting no DM DX.

#### Algorithms

We experimented with two statistical learning algorithms: LogisticRegression (a linear regression for classification tasks) and XGBClassifier (an algorithm that trains multiple decision tree classifiers and combines the results, referred to as gradient boosting). We used the default logistic regression implementation from the sklearn Python library and the XGBoost library implementation of the XGBClassifier.

#### Input time window lengths

We also experimented with increasing lengths of input time windows to understand whether the predictive ability of statistical learning models changes with longer time windows (that is, as they evaluate data collected from longer time periods, instead of carrying out an independent test on each day separately). We tested the two algorithms with 5 different input time window lengths (1-, 3-, 7-, 14-, and 21-night windows). We extracted rolling, overlapping windows with a 1-day stride for all input sizes to identify all possible sample time windows. To do this, we shifted the window forward by 1 night from its previous position to extract each new sample. Samples were excluded if more than 2 nights in each week were missing (so valid samples included ≤2 nights missing for 3-night windows, ≤2 nights missing for 7-night windows, ≤4 nights missing for 14-night windows, and ≤6 nights missing for 21-night windows). This ensured similar data quality as the window size changed. Further, we identified the start dates for the 21-night windows that passed this criterion and, for datasets of all other window lengths, only used samples that started on these start dates. Thus, all models, each a combination of different window lengths and algorithms, were tested on the same set of individuals and start dates of time windows. By varying the length of the input time windows, we assess when the predictions become stabilized and how much having more nights of data (i.e., longer time windows) improves predictions.

#### Dataset splitting

We split our controlled dataset into 80% training/validation data and 20% held-out testing data by individual, with a balanced number of individuals self-reporting DM and individuals self-reporting no DM DX, as well as a balanced number of individuals in each age bin in each dataset. We report the number of individuals and periods from individuals split by dataset and label in Table 3. We optimized hyperparameters with 3-fold cross-validation prior to the final evaluation of our algorithms on the held-out testing data. Optimized hyperparameters were number of estimators = 200, learning rate = 0.01, and max depth = 3 for the XGBClassifier and *C* = 0.01 regularization strength and L2 regularization penalty for the LogisticRegression algorithm across all lengths of input time windows.

**Table 3:** Dataset splits for machine learning training, validation, and testing

	Num. Individuals			Num. Windows from Individuals		
	Rep. No DM DX	Rep. DM	Total	Rep. No DM DX	Rep. DM	Total
**Train/Val**	226	189	**415**	17,281	8481	**25,762**
**Test**	56	47	**103**	3823	2140	**5963**
**Total**	**282**	**236**	**518**	**21,104**	**10,621**	**31,725**

Dataset split into training/validation and testing data after filtering for machine learning. Subtotal rows and columns are bolded. People rep. DM = people who self-reported diabetes mellitus, People rep. No DM DX = people self-reporting no diagnosis of diabetes mellitus.

#### Assessed metrics for evaluating performance

For each combination of classifier and input time window, we report the Receiver Operating Characteristic (ROC) Curve with Area Under ROC (AUROC) and the Precision-Recall Curve (PRC) and Area under PRC (AUPRC) metric. We report True Positive Rate (TPR), False Positive Rate (FPR), precision, recall, sensitivity, specificity, and F1 score at the optimal threshold selected using Youden’s J statistic (J = Sensitivity + Specificity—1 = TPR—FPR) in Supplementary Table 5. We select the optimal model based on AUROC and AUPRC because we analyze the probabilities outputted by the model in later analyses. AUROC and AUPRC consider these probabilities, whereas metrics like F1 score convert the probabilities to a binary variable based on a specific threshold, reducing the information considered in the metric.

### Analysis of other chronic conditions

Other chronic conditions also perturb the physiological data streams we measured, so we suspected that people reporting no DM DX with other chronic conditions might be falsely predicted as more likely to be people self-reporting DM. The other chronic conditions we collected data on are: hypertension, coronary, myocardial infarction, Congestive heart failure, stroke, Atrial fibrillation, sleep apnea, COPD, asthma, cancer, anemia, and immunodeficiency. We also collected plain text responses about other respiratory conditions. If an individual reported that a healthcare professional had diagnosed them with any of these conditions, we categorized them as having an “other chronic condition”. We extracted the average predicted probability of each individual and compared individuals self-reporting no DM DX with any chronic condition against individuals self-reporting no chronic conditions. We compared quantiles to assess the difference in distribution between the average predicted probabilities of individuals self-reporting no DM DX with any chronic condition and the average predicted probabilities of individuals self-reporting no chronic condition. We analyze the predictions from one of the top-performing algorithms (21-night XGBClassifier). We plotted a box plot of the predictions from the population of individuals self-reporting no DM DX who reported another chronic condition (e.g., Hypertension, Sleep Apnea, Cancer) against the population self-reporting no chronic conditions. We included a box plot of the predictions of the population with DM for visual comparison. We also conducted a Kruskal–Wallis test to test whether there was a significant difference between the three groups, followed by Posthoc Dunn’s Test, 2-sided. For Dunn’s Test, *p*-value significances were adjusted using the Benjamini/Hochberg *p*-value threshold correction. All these analyses were performed with our testing dataset.

### Feature importance and ablation

We evaluated which features contributed most to predictions. Both the algorithms we used, LogisticRegression and XGBClassifier, have a standard method to quantify feature importance, or how much each feature contributes to predictions. We extracted these quantities from our best-performing statistical model (21-night XGBClassifier) and aggregated them over time. Results are shown in Fig. 4a.

Another method for evaluating the value of features in performance is feature ablation. To test for this, we grouped our features into 5 different feature sets, shown in Table 2. We then generated all possible permutations of these sets, where the length of each permutation ranged from 1 to 5 sets. With the best performing model, we observed performance as we reran the model for every possible combination of feature sets. Two different orders for adding features to length 3 would be 123 and 231, and two different orders for adding features to length 4 would be 1234 and 4312. We expected to see an improvement in performance as we added features derived from different data streams and more complex techniques. For every feature set, we observe the change in performance as we move from a combination of length n that doesn’t contain the set, to a combination of length n + 1 that is identical to the original combination except for the addition of the given set at the end. Through this method, we hoped to gain an understanding of how much each set of features contributes to the accuracy of predictions. We display the mean increase (over 16 feature addition combinations) in AUROC as a result of adding each feature set in Fig. 4b.

We also performed the analysis of other chronic conditions (see *Analysis of Other Chronic Conditions* in Methods for more detail) on feature-ablated models, to see if adding certain groups of features improved the distinction in predictions between people self-reporting DM, people self-reporting no DM DX with any other chronic condition, and people self-reporting no chronic conditions. We used the Kruskal–Wallis test, followed by Dunn’s test (with Benjamini/Hochberg p-value threshold correction), to compare the groups derived from using only feature sets 1–3 as opposed to using feature sets 1–5. In addition, we also used the Mann–Whitney U test to compare each group to its counterpart that was derived using the other group of feature sets. Results are shown in Fig. 4c. We used Kruskal–Wallis tests and Mann–Whitney U tests instead of ANOVAs and t-tests to avoid assumptions of normality in the tested distributions. We excluded feature sets 4 and 5 because our ablation experiments showed that excluding these features led to substantial differences in separability amongst the Analysis of Other Chronic Conditions groups. Moreover, these feature sets are both derived from the temperature sensor, because many wearable health devices do not have access to this sensor, we wanted to see how the exclusion of features from this sensor affected performance. Distal body temperature is a more stable physiological indicator than HR, so we hypothesized that its inclusion had contributed to novel separation of physiology consistent with DM with higher specificity. Additionally, many wearable devices still do not have temperature sensors, and so removing these features allows us to examine how well devices without temperature data might fare on these tasks. All these analyses were performed with our testing dataset.

### Evaluation of best model in an imbalanced cohort

Previously, our analyses of the balanced cohort indicated that longitudinal data and secondary features of sleep and distal body temperature improved the algorithm’s identification of physiology consistent with DM. However, performance in the balanced cohort does not directly represent performance in the general population, where there is an imbalance between people self-reporting DM and people self-reporting no DM DX. So, we additionally evaluated the best model in an imbalanced cohort to demonstrate its performance within a general population. The exact proportion of individuals with DM varies from country to country, so we chose to match the US distribution of people with DM (17.9% as estimated by the AHA^1^). Metrics that depend on the proportion of each class (e.g., AUPRC) should be expected to vary in populations with different proportions of people with DM. Further, this model is not tuned for optimal performance, so the outcomes of this experiment should not be considered the maximal performance that could be achieved in the general population. These outcomes represent the degree to which this model improves the separability of physiology consistent with DM in a general population. We discuss methods to optimize this model in the discussion.

This model was trained on the training and validation data from the balanced cohort and evaluated using a combination of the testing data from the balanced cohort and additional data from individuals excluded when constructing the balanced cohorts. Exact cohort sizes for the training data are identical to those of the balanced cohort (Table 3; row “Train/Val”).

**Fig. 5:**
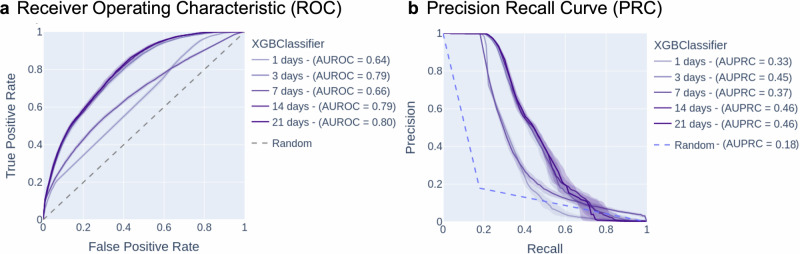
Evaluation of the best model on an imbalanced cohort. ROC Curve (**a**) and PRC Curve (**b**) of best algorithm (21-night XGBClassifier) on the imbalanced cohort with a class balance of 17.9%. Results are the average of 100 repeated trials where individuals are randomly drawn from a pool of 6571 individuals who self-reported no DM DX. Shaded regions indicate the interquartile range of repeated experiments at each threshold.

The following describes the evaluation of the trained model. From the 9378 individuals for whom we successfully extracted features, we extracted varying-length time windows and applied data quality standards. Notably, while the majority of individuals were excluded in order to balance the classes, some individuals were excluded to balance age and sex, and rebalancing in the larger dataset led to an increased number of individuals self-reporting DM in the testing data. The imbalanced cohort testing dataset consisted of 87 individuals who self-reported DM and 6571 individuals who self-reported no DM DX, not in the training dataset. For each repetition, we randomly sampled 399 individuals who self-reported no DM DX, balanced by age and sex, to obtain a 17.9% class balance in the testing dataset. We repeated our random sampling of individuals 100 times and showed the variance of the ROC and PRC curves (interquartile range (IQR)) in Fig. 5. We randomly sampled 10 windows from each individual to preserve the class balance. So, each curve was generated from 870 windows from people self-reporting DM and 3990 windows from people self-reporting no DM DX. Results are shown in Fig. 5, and all metrics are shown in Supplementary Table 6.

### Statistics and reproducibility

Here, we will summarize the statistical methods used across our manuscript. The statistical testing for differences in individual features is described in *Feature Extraction > Statistical Testing for Differences in Individual Features*. For each feature, we used the Mann–Whitney U test for difference in distribution, 2-sided, to test whether and 24,486 nights from people self-reporting DM differed from 764,315 nights from people self-reporting no DM DX (more detailed sample sizes in Table 1).

The statistical testing for the analysis of other chronic conditions is described in the *Analysis of Other Chronic Conditions* section. We conducted a Kruskal–Wallis test to test whether there was a significant difference between the three groups, followed by Posthoc Dunn’s Test. For Dunn’s Test, *p*-value significances were adjusted using the Benjamini/Hochberg p-value threshold correction. Both tests used 47 people self-reporting DM and 38 people self-reporting no DM DX with no chronic conditions, and 18 people self-reporting no DM DX with another chronic condition (more detailed sample sizes in Supplementary Table 1). For the analysis of true positive, false negative, and true negative time windows, we used 3093 true negatives, 394 false negatives, and 1746 true positive time windows to conduct a Kruskal–Wallis test, followed by a 2-sided Posthoc Dunn’s Test. Detailed information is in Supplementary Table 2. The statistical testing for differences between feature-ablated models within chronic condition groups is described in *Feature Importance and Ablation*. We used the Kruskal–Wallis test, followed by the 2-sided Dunn’s test (with Benjamini/Hochberg p-value threshold correction), to compare the groups derived from using only feature sets 1–3 as opposed to using feature sets 1–5. Finally, we also used the Mann–Whitney U test, 1-sided, to compare each group to its counterpart that was derived using the other group of feature sets. We used Kruskal–Wallis tests and Mann–Whitney U tests instead of ANOVAs and t-tests to avoid assumptions of normality in the tested distributions.

## Results

### Availability of Collected Data

We obtained physiological, sleep, and self-report data from 33,374 individuals through the TemPredict study^54^, of whom 389 self-reported having received a DM DX from a healthcare professional (1.16%), 10,820 self-reported having never received a DM DX from a healthcare professional (32.4%), and 21,402 individuals did not self-report this information (64.1%; see Fig. 1a). The wearable devices (Oura Ring) collected physiological metrics including HR, HRV, distal body temperature, respiratory rate, and respiratory rate variability data between April 22nd, 2020 and August 29th, 2020 (4 months and 7 days). We obtained nightly-level physiological data of HR, HRV, respiratory rate, and respiratory rate variability that were generated by the proprietary Oura algorithm. We also obtained both nightly-level sleep/wake onset data and sleep stage data predicted every 30 seconds from the proprietary Oura algorithm^45–47^. We finally obtained a minute-level data stream of distal body temperature. We extracted nightly summary features of the distal body temperature data and the sleep stage data (see “Methods”, Feature Extraction). We used nightly summary features (Fig. 1b) for analyses. After data filtering, we obtained 24,486 nights from 315 people self-reporting DM and 764,315 nights from 9063 people self-reporting no DM DX with 36 features, 84.3 nights per individual (65.3% data completeness). This corresponds to 84.6 nights per individual (65.6% data completeness) from individuals who self-reported no DM DX and 77.7 nights from individuals (60.2% data completeness) who self-reported DM. Data completeness was calculated as a fraction of nights data was collected for (129 nights). Age and sex data are reported in Table 1.

### Individuals who self-reported DM differ from individuals who self-reported no DM DX in commonly used features that are representative of noninvasive physiological data streams at a longitudinal, population level

We first selected four “commonly used features” that closely represent changes within a night in a physiological data stream at the nightly level and are commonly used as indicators for a variety of remote health monitoring and wellness settings^31–33,35–38,45,49^. We refer to average resting HR, average resting HRV, TST, and DDBTA as the commonly used features (see “Methods”, Feature Extraction). Figure 1b shows the feature extraction data pipeline. We then tested whether the commonly used feature values of the population self-reporting DM differ from those of the population self-reporting no DM DX.

Figure 1c–f show the differences in commonly used feature values between the population self-reporting DM and the population self-reporting no DM DX, separated by age group within each population. The older population self-reporting no DM DX had lower HRV than the younger population self-reporting no DM DX. All the age groups self-reporting no DM DX had similar kernel density estimates, and distributions of groups self-reporting DM were consistently shifted away from the blue lines. The darker red lines that represent the older population self-reporting DM tend to be less Gaussian across the figures (e.g., Fig. 1f: both the 60–69 and 70–79 distributions are bimodal; similar pattern in Fig. 1c), suggesting more variability in the older population self-reporting DM than the older population self-reporting no DM DX.

The commonly used feature values from the population self-reporting DM differed significantly from those of the population self-reporting no DM DX in HR, RMSSD, TST, and DDBTA (Mann–Whitney U test for difference in distribution; 2-sided; *p*-value < 0.0001 for all tests; test was repeated for all features, within each age group (*n* = 764,315 nights from people self-reporting no DM DX; *n* = 24,486 nights from people self-reporting DM) and across all age groups (sample sizes per age group in Table 2)). Across age groups, Cohen’s *d* between people self-reporting DM and people self-reporting no DM DX were as follows: HR = 0.607, RMSSD = −0.555, TST = −0.192, DDBTA = −0.455, suggesting that more nuanced features or algorithms that use combinations of features could detect the effects of DM. We report the mean and standard deviation of the population self-reporting DM and the population self-reporting no DM DX for each feature and the effect size (Cohen’s *d*) between the feature distributions of the population self-reporting DM and the population self-reporting no DM DX in Table 4. We extracted 32 other features (see *Feature Extraction* in “Methods”) from prior work and found significant differences in these features as well (shown in Supplementary Table 4).

**Table 4:** Statistics for common feature values of people self-reporting DM and people self-reporting No DM DX

Feature	Measures of central tendency	People rep. no DM DX	People rep. DM	Effect Size: Cohen’s d (Mann–Whitney U P < 0.0001 for all)
**All**	**Num. Nights**	764,315	24,486	-
**Heart rate average (BPM)**	**mean**	62.713	67.819	0.607
	**std**	8.852	9.569	
**Root mean square of successive difference (ms)**	**mean**	42.944	30.141	−0.555
	**std**	24.973	17.180	
**Total sleep time (hrs)**	**mean**	7.946	7.649	−0.192
	**std**	1.726	1.990	
**Distal body temperature circadian amplitude (°C)**	**mean**	7.261	6.008	−0.455
	**std**	2.848	2.438	

Mean and standard deviation for the commonly used features around heart rate, heart rate variability, and sleep efficiency. Statistics were calculated using features extracted at the nightly level. All shown effect sizes differed significantly (*p* < 0.0001; Mann–Whitney U test for difference in distribution; 2-sided; sample sizes are “Num. Nights”).

*BPM* Beats per minute, *ms* milliseconds, *BrPM* Breaths per minute, *°C* Degrees Celsius, *hrs* hours, *std* standard deviation, *People rep. DM* people who self-reported diabetes mellitus, *People rep. No DM DX* people self-reporting no diagnosis of diabetes mellitus.

### Machine learning algorithms predict whether multiple-night time windows of physiological features are drawn from a person self-reporting DM or a person self-reporting no DM DX

We aimed to improve upon the longitudinal, population-level differences that we found initially by testing whether multiple-night time windows drawn from individuals could be classified as having been drawn from a person self-reporting DM or a person self-reporting no DM DX. We trained LogisticRegression and XGBClassifier algorithms on input time windows of varying numbers of nights, resulting in 10 trained machine learning models (see *Machine Learning* in Methods). Figure 2 shows these models’ results on the held-out testing data, 5963 time windows drawn from 103 individuals (see Table 3 for detailed sample size data).

The Receiver Operating Characteristic (ROC) curves and Precision-Recall Curves (PRC) obtained from these models show that the algorithms can be tuned to obtain a range of strong performances. All of the classifier performances, as determined by Area Under the ROC Curve (AUROC) and Area Under the PRC (AUPRC), are well above 0.5, demonstrating that classification is feasible in this context. The best performing algorithms were the 14- and 21- night XGBClassifier models (AUROC = 0.88, 0.88; AUPRC = 0.79, 0.80). Figure 2b, d show the AUROC performance of all 10 algorithms (range from 0.73 to 0.88), and Fig. 2c, e show the AUPRC performance of all 10 algorithms (range from 0.67 to 0.80). These results show that multiple-night time windows can nonrandomly be classified as coming from a person self-reporting DM or a person self-reporting no DM DX (AUROC > 0.5; AUPRC > 0.5) based on combinations of physiological features extracted during that time window. We further illustrate the performance of our best model (21-night XGBClassifier) in Fig. 3a. Out of 3823 time windows from individuals self-reporting no DM DX, 394 windows were predicted to be drawn from an individual self-reporting DM, indicating a FPR of 19.1%. Out of 2140 time windows from individuals self-reporting DM, 1746 windows were predicted to be drawn from an individual self-reporting DM, indicating a TPR of 81.6%.

### Machine learning algorithms reach best performance after at least 14 nights are included in input time windows

We hypothesized that the effects of DM on physiological data streams may not be present consistently each night because DM can vary over time, but that physiologically aberrant days should be more common in individuals with chronic conditions. If this is correct, then longer time windows with more nights of data would be more likely to accumulate consistent feature differences between people self-reporting DM and people self-reporting no DM, DX vs. different-length time windows showing relatively similar performance. Figure 2b–e show that AUROC and AUPRC improve as input time windows increase in length, in both algorithms, on the held-out testing data (5963 time windows drawn from 103 individuals; see Table 3 for detailed sample size data). The 1-night models perform worse (LogisticRegression: AUROC = 0.73, AUPRC = 0.67; XGBClassifier: AUROC = 0.74, AUPRC = 0.67) than all other models of the respective algorithms. The 3- and 7-night models perform slightly worse than the 14- and 21- night models (LogisticRegression, AUROC: 0.87 < 0.88; XGBClassifier, AUROC: 0.84–0.86 < 0.88; AUPRC: 0.76–0.77 < 0.79–0.80). The 14- and 21-night models have the same AUROC and AUPRC in all models except for one slight discrepancy (XGBClassifier AUPRC; 0.79 vs 0.80). Metrics for the best threshold on the ROC curve are shown in Supplementary Table 5. These results confirm that algorithm performance improves with longer time windows until at least 14 nights are included in the input time window.

### People self-reporting no DM DX with other chronic conditions did not show significantly different predicted probabilities compared to those self-reporting no chronic conditions

Our testing data contained 18 individuals self-reporting no DM DX while reporting a chronic condition other than DM (18 out of the 56 individuals self-reporting no DM DX in the test dataset). Some of the physiological effects of DM are likely shared by other chronic conditions (e.g., hypertension, sleep apnea), so we assessed the degree to which time windows from individuals self-reporting no DM DX with other chronic conditions are incorrectly predicted to be drawn from people self-reporting DM by the best model identified previously. Figure 3b shows distributions of the average predicted probability of DM for each individual within each group (people self-reporting DM, people self-reporting no DM DX with any other chronic condition, and people self-reporting no chronic conditions). The box plots in Fig. 3b show that the people self-reporting no DM DX with another other chronic condition have lower predicted scores than people self-reporting DM, and predicted scores similar to those of individuals self-reporting no chronic conditions. We observed that the distributions of predicted probabilities of the two cohorts of people self-reporting no DM DX (with and without other chronic conditions) were both significantly different from the distribution of predicted probabilities for the cohort self-reporting DM (Posthoc Dunn’s; 2-sided; People Self-Reporting DM vs. People Self-Reporting No Chronic Conditions, *p*-value = 6.9e-12; People Self-Reporting DM vs. People Self-Reporting No DM DX with Other Chronic Conditions, *p*-value = 7.0e-6; see Supplementary Table 1 for all *p*-values). At the same time, the probabilities of the cohorts self-reporting no DM DX were not significantly different from each other. These results indicate that people self-reporting no DM with any other chronic condition did not show significantly higher predicted probabilities than individuals self-reporting no chronic conditions.

### The commonly used features of false negative time windows resemble those of true negative time windows more closely than those of detectable time windows drawn from people self-reporting DM

We observed that the best model’s TPR (81.6%) appeared relatively low, prompting further exploration. We assessed our models’ false negative predictions by exploring the values of the commonly used features supported by prior work to be relevant to the effects of DM^31–33,35–38,45,49^. If commonly used representations of these data streams from false negative windows resemble those of a healthy population, it suggests that some time windows from people self-reporting DM cannot be determined to be from a person self-reporting DM with the collected data streams. We grouped the model’s predicted probabilities on the held-out testing data (5,963 time windows drawn from 103 individuals; see Table 3) into the true positive group, false negative group, and true negative group. We compared the commonly used features of the three groups to understand whether the false negative group resembled the DM-negative population (true negative) more than the population with detectable DM (true positive). Figure 3c shows four subfigures, where each subfigure shows predicted probabilities against a commonly used feature. In each subfigure, the points in light blue represent the false negative predictions, and the dark blue vertical lines represent the median (solid) and quartiles (dashed) of the true negative predictions. The red points represent the true positive predictions. Following a significant Kruskal–Wallis test comparing the false negative, true negative, and true positive groups for each commonly used feature (HR: *p*-value = 4.2e-5; RMSSD: *p*-value = 4.9e-6; TST: *p*-value = 4.7e-3; DDBTA: *p*-value = 1.2e-4), we compared each pair of groups with 2-sided post hoc Dunn’s tests (see Supplementary Table 2). True negatives were significantly different from true positives in all cases. By contrast, true positives were not significantly different from false negatives in three of the four commonly used features (HR, TST, DDBTA). This supports the hypothesis that the low TPR resulted from some time windows from people self-reporting DM not being discernable from the general population based on our data streams. We expand on limitations due to factors related to our model’s TPR in the discussion.

### Features of HR and HRV were the most important in making predictions

Identifying the most informative features can reveal which sensors in a wearable device are likely to be more useful for the detection of physiology consistent with DM, as well as provide direction for better feature engineering approaches to strengthen future models. We examined the importance of individual features by assessing both internal classifier parameter values and the change in AUROC and AUPRC on the testing data after varying the input features to the best algorithm, which was subsequently retrained on altered versions of the training data (See Feature Ablation in “Methods”). As shown in Fig. 4a, the highest average feature importance for individual features was found in two items in feature set 1 (hr_lowest, rmssd), demonstrating that features derived from HR and HRV were the most informative in identifying people self-reporting DM out of all the features used in this study (see Supplementary Table 3 for all importance values). Similarly, Fig. 4b shows that in an analysis of which feature set led to the largest increase in AUROC when added to the input, feature set 1 is one of the top two sets. These results suggest that HR- and HRV- related features are important for DM detection-related tasks with noninvasive physiological data.

### Distal body temperature features improved separation of individuals with other chronic conditions than DM

In a similar effort to identify important sets of features that contribute most to accurate predictions, we identified that distal body temperature-related features held high importance. Figure 4a reveals that four of the secondary temperature features were among the most important (sleep_mean, sleep_wake_difference, diurnal_distal_body_temp_amp, complexity_0). Inclusion of feature set 5 (secondary temperature features) increased AUROC more than any other set (Fig. 4b); primary temperature features (set 4) do not contribute to increases in AUROC as much as the secondary features (set 5). This difference between the importance of the primary and the secondary distal body temperature features suggests that features related to CI and diurnal amplitudes (here referring to differences between night and day) of distal body temperature are useful for such detection tasks.

We hypothesized that predictions of time windows from people self-reporting no DM DX with other chronic conditions than DM may vary with ablated features. We found that the difference in average predicted probability between these two groups (people self-reporting no DM DX with and without other chronic conditions) differed by less than 10% at all quantiles across single-feature ablation trials. We further tested whether the model’s ability to distinguish people self-reporting no DM DX with other chronic conditions from people self-reporting DM varied when we changed which sets of features were included in model training, and we found that the distal body temperature features (feature sets 4 and 5) led to substantial differences.

We subsequently tested whether distal body temperature features contributed to higher specificity with an analysis comparing two models trained on altered versions of the training data: one model trained on feature sets 1, 2, and 3 and one model trained on all 5 features sets, i.e., adding the two temperature feature sets. Figure 4c shows the average predicted probabilities of these two models for each of the analysis of other chronic condition groups (people self-reporting no chronic conditions, people self-reporting no DM DX with other chronic conditions, and people self-reporting DM) on the testing data. Statistical tests for difference in distributions showed that the distribution of predicted probabilities from the model trained using feature sets 1, 2, and 3 differed significantly from the distributions of predicted probabilities from the model trained using feature sets 1, 2, 3, 4, and 5 (Mann–Whitney U, 1-sided; people self-reporting no chronic conditions: *n* = 38, *p*-value = 0.0005; people self-reporting no DM DX with other chronic conditions: *n* = 18, *p*-value = 0.100; people self-reporting DM: *n* = 47, *p*-value = 0.014). The model that is trained without the primary and secondary distal body temperature features yielded substantially higher predicted probabilities for people self-reporting no DM DX with other chronic conditions (0.082 higher), which reduces the ability of such models to correctly distinguish between people self-reporting no DM DX with other chronic conditions and people self-reporting DM. This finding supports the hypothesis that distal body temperature, as a more stable physiological indicator, contributed to higher separation of physiology consistent with DM with higher specificity.

In all three groups, the addition of temperature features resulted in a higher probability for the cohort self-reporting DM to be predicted as DM-positive and a lower probability for the two cohorts self-reporting no DM DX to be predicted as DM-positive. We also compared the predicted probabilities of all of the cohorts (Posthoc Dunn’s, 2-sided, see Supplementary Table 1). In both models, when using only the first three features as well as using all five features, the predicted probabilities of both cohorts self-reporting no DM DX were significantly different from the predicted probabilities of the cohort self-reporting DM, but were not significantly different from each other. We observed better differentiation when all five feature sets were used. These results demonstrate that we were able to differentiate people self-reporting DM from people self-reporting no DM DX, regardless of whether they had other chronic conditions, and that this differentiation was strengthened by the addition of temperature features.

### Best model performs comparably in an imbalanced cohort matching a general population

Performance in a balanced cohort does not directly represent performance in the general population, where there is an imbalance between people self-reporting DM and people self-reporting no DM DX. So, we additionally evaluated the best model on an imbalanced cohort to demonstrate its performance within a general population. We evaluated the trained model from the balanced cohort analyses (cohort sizes in Table 3; row “**Train/Val**”) on a newly constructed imbalanced dataset. This imbalanced dataset was constructed with all possible individuals self-reporting DM not in the training data (*N* = 87) and a random sample (*N* = 399) drawn from 6571 individuals self-reporting no DM DX, set to match the US distribution of people with DM (17.9% as estimated by the AHA^1^). We repeated the random sampling 100 times and showed the IQR in Fig. 5. As shown in Fig. 5, the 21-day XGBClassifier achieved a median AUPRC of 0.46, which represents a 0.28 improvement over a random classifier (0.179 AUPRC). See Supplementary Table 6 for all metrics reported at the optimal threshold. This improvement over a random classifier is comparable to that of the analysis in the balanced cohort, where the model’s AUPRC on the balanced cohort (0.80) represented a 0.30 improvement over a random classifier. The optimal threshold on this curve achieves sensitivity = 80.6% and specificity = 63.1%. This indicates that the model identifies a similar amount of nonrandom information as in the previous balanced cohort analysis, even when applied to an imbalanced dataset drawn from a much larger population.

## Discussion

The work we present here supports the feasibility of detecting individuals with physiology consistent with DM using noninvasive wearable data without glucose data. Commercially available wearable devices continuously monitor data streams related to physiology (e.g., HR, HRV, sleep, and distal body temperature), and researchers may be able to use these data to measure the physiological effects of DM and/or related conditions on the cardiovascular and autonomic nervous systems, as well as the effects of DM on sleep. Unlike in the detection of acute infections, for chronic conditions, the physiological changes might be relatively small but persistent, and so easier to separate by resampling over time. We hypothesized that signatures of these effects could be identified based on a combination of features extracted from the different data streams over sufficiently long periods of time. Individuals self-reporting DM differed significantly from individuals self-reporting no DM DX in average nightly HR, average nightly HRV, TST, and DDBTA with moderate to large effect sizes. Given these differences, we extracted 36 features of the physiological signatures of DM and tested whether machine learning algorithms using this larger feature set could detect when multiple-night time windows were drawn from an individual self-reporting DM. The gradient boosting model (XGBClassifier) achieved the best performance with AUROC = 0.88 and AUPRC = 0.80. Longer periods of time increased the model’s sensitivity to differences between the two classes (AUROC = 0.74 [1-night] vs. 0.88 [14-nights]). Analysis of other chronic conditions showed that the model effectively separated DM from other chronic conditions (i.e., self-reporting other chronic conditions did not increase false discovery). Feature importance analyses revealed that HR/HRV features (set 1) and secondary distal body temperature features (set 5) impacted performance the most (HR/HRV: delta AUROC = 0.0729; distal body temp.: delta AUROC = 0.0724). We further found that excluding primary and secondary temperature features (sets 4 and 5) increased the likelihood of misclassifying other chronic conditions as DM. These findings evinced the value of longitudinal data and the secondary features for identifying physiology consistent with DM.

Finally, we demonstrated the performance of the model on an imbalanced dataset emulating a general population. We expected with a larger, heterogeneous, negative population, more false positives should arise because people differ biologically^55^. Across 100 repeated trials with data drawn from 87 individuals self-reporting DM and 6571 individuals self-reporting no DM DX, the model demonstrated an average AUROC = 0.80 and average AUPRC = 0.46 (0.28 improvement from a random baseline with 0.179 AUPRC). This matched our expectations. Nevertheless, it recovers comparable nonrandom information in an imbalanced cohort to the balanced cohort, indicating the robustness of sleep and distal body temperature features and highlighting the importance of measuring across time and not relying on single time points or days. These findings support the feasibility of developing systems for outpatient monitoring that could screen individuals who merit clinical follow-up for suspected DM using physiological data from common wearable devices before individuals might otherwise have reasons to seek out and use continuous glucose monitors.

Next, we will discuss how our work compares to prior literature, addressing three distinct gaps. First, where prior work typically uses less than a day of input^5,22–32^, we assess the impact of longitudinal input windows ranging up to multiple weeks and demonstrate improved sensitivity with longer windows (AUROC increases by 0.15 from 1-night to 21-night models, in balanced and imbalanced cohorts). Second, prior work was generated from small cohorts (the largest cohort of 70 individuals), that raise questions of the generalizability of their findings^5,22–32^. Our work tests the feasibility of detection physiology consistent with DM in much larger cohorts: initially testing feature differences with 9378 individuals (788,801 nights), then testing machine learning models with 518 individuals (31,725 time-windows), and finally assessing model performance with 6,658 individuals. Third, prior work chiefly assessed statistical aggregation functions of wearable data streams (primary features)^5,26–32^, which are nonspecific to the effects of DM within data streams that are constantly fluctuating in natural settings. Detection methods relying on statistical features are likely to raise numerous false alarms in natural settings. Our work demonstrates in naturalized contexts that features derived to capture biological dynamics (secondary features) lead to gains in separability of physiology consistent with DM (delta AUROC = 0.09), complementary to primary features. Secondary features are likely also essential to developing highly specific algorithms for other tasks requiring identification of physiology consistent with DM in natural settings.

Several prior works assessed whether features of HR, HRV, movement, EDA, and in some instances, distal body temperature could identify time-specific DM-related events^29–32^.

Time-specific aberrations in physiological data were associated with, and used to predict changes in, CGM data or hypoglycemic events, which our work notably did not have access to. Other prior work investigated specific secondary features but only tested for statistical differences, reporting metrics of effect size that don’t directly indicate the efficacy of algorithms that combine several such secondary features with primary features^35,38,39^. Though interesting, neither literature was able to test their efficacy in large, real-world cohorts representing a practical assessment.

Our work showed that the methods we assessed (repeated measures over time and diverse featurization) increased model robustness in a large-scale cohort of natural wearable data. Our imbalanced analyses point to the need for further research into the kinds of biological heterogeneity interfering with detection. While prior work reported higher performance, our data is more representative of real-world performance as we captured a broader population segment with our larger cohorts (balanced cohort is 5× and imbalanced cohort is 91× the largest prior cohort of wearable data)^5,29–32^. Our large-scale analyses indicate much higher variability in the general population. Practical assessments, as we showed in this report, are needed to identify and overcoming barriers in diverse, general populations and natural contexts. Large, publicly available databases of wearable and CGM data are essential to facilitate practical assessments and accelerate the translation of wearable health tools to the real world.

Next, we will discuss several directions for future work that our analyses and findings support. Improved model performance from longer periods of input suggests that longitudinal studies using noninvasive data are better equipped to detect DM than short studies of a few minutes of data. The importance of both the sleep and temperature features suggests the utilization of wearable devices that are capable of measuring both for future studies, as they substantially informed DM detection. Diurnal distal body temperature amplitude was used to characterize circadian rhythm stability; since DM can affect circadian rhythm stability^51^, this feature was a useful differentiator of people who have DM from those who do not. Future work may consider circadian patterns in HR, which we could not explore due to high missingness in daytime HR data. Similarly, the complexity features, which represent characteristics of the fluctuations of the minute-level distal body temperature time series, may have provided insight into how DM affects people’s nocturnal thermoregulation^6^ through its impact on the autonomic nervous system (see *Feature Extraction* in “Methods”). Finally, the confidence gained in detections from repeated measures over time and features capturing a breadth of physiological effects may make possible the detection of other chronic conditions that affect people’s sleep physiology.

Noninvasive wearables equipped with high specificity algorithms may 1 day function in tandem with CGM devices to reduce the burden of glucose sensing. Noninvasive methods relying on HR and HRV lack specificity because HR is such a sensitive measure to changes in the body. As a result, algorithms developed to use these features in combination with periodically sampled CGM measurements (in order to detect hypoglycemic events in between CGM sampling) are limited by the specificity of the noninvasive methods (false alarms of the system will be proportional to the false alarms generated by the noninvasive component). Our feature importance analyses indicated that secondary features of distal body temperature and sleep elucidate more specific information to DM than the primary features for our task. Future work should test whether the secondary feature of distal body temperature and sleep shows comparable improvements in specificity when applied to the more challenging tasks of hypoglycemic event detection and glucose value prediction. Positive findings of such assessments would directly suggest the feasibility of high specificity monitoring of DM in between CGM measurements.

Our analyses were performed with nightly summary features, congruent with features used in prior work^56–58^. But in the future, higher granularity features could be explored. Higher granularity in the temporal domain could be leveraged to better capture the dynamics of underlying physiology, such as the more detailed behavior of the distal temperature complexity. Our study does not explore how cross-data stream temporal dependencies between features might relate to the presence of DM in an individual. In addition, longer time horizons than 21-days contain more information but more complex longitudinal patterns. We chose this length of time (1) so that several windows per individual would pass our data availability inclusion criteria and (2) to minimize the effects of behavioral variability and intrinsic physiological cycles (e.g., weekday-weekend patterns, menstrual cycling); two factors whose effects on longitudinal wearable data have not yet been well studied. Promising directions for future work would be to assess the value of greater longitude, higher-granularity temporal dynamics, and cross-data relationships, especially using wavelet analysis or graph-based approaches.

Finally, we will discuss limitations to our approach. First, our approach is limited to modalities measured by the wearable device used. Other wearable devices with different sets of modalities may change the utility of this approach, especially since different sensors have different margins of error in measurement. Second, sensor accuracy can vary with skin tone and ethnicity. Our population was majority white (83.55%; see Supplementary Table 7), so we lacked the diversity to appropriately account for this variable. Third, our ground truth labels are self-reported, which likely carries some imprecision, and they do not distinguish between types of DM, suggesting some of our time windows may not have been representative of their class. Fourth, we did not explore deep learning algorithms as there are numerous combinations of architectures and training methods, and differences between these combinations don’t meaningfully test our hypotheses regarding sleep and skin temperature features. Fifth, the deleterious physiological effects of hyperglycemia vary within an individual over time, based on environmental factors, as well as across individuals, possibly based on genetic factors. The variance in physiological effect unavoidably limits the effectiveness of any physiologically based detection tool working in isolation. We attempted to account for these limitations by studying false negative time window predictions. We studied time windows from individuals' self-reporting DM, within which the features did not reflect the effects of DM. If the commonly used features of false negative windows resemble those of a healthy population, then the model might be limited in that some time windows from individuals' self-reporting DM may not reflect the effects of DM based on our collected data streams. We found that the commonly used features of the false negative predictions were more similar to those of the true negative distributions than to those of the true positive distributions, indicating that some time windows from people self-reporting DM may not be discernable from the general population with the data streams we obtained. This is consistent with some individuals managing their DM more effectively at some times than others, as might be expected. For this reason, a real-world screening system using approaches like those we evaluate here would likely benefit from repeated re-testing over time before alerting individuals of potential risk for DM. We lack the data to assess that assertion here.

While we attempted to account for seasonal changes in environmental temperature by limiting our data to span a single season (spring), we couldn’t account for climate differences between geographical areas. Our data is gathered from all over the US and so represents a variety of climates with differing environmental temperatures. Future work may analyze the relationships between colder environmental temperature and longitudinal wearable data and produce methods to optimize the wearable detection algorithms across climates. Another limitation to our findings is that they cannot be reproduced due to the data being private commercial data that is only available upon a reasonable or qualified request. Policies to improve data sharing from devices to users would likely make research of using device data more reproducible and impactful. Finally, we note that a wide breadth of behavioral and health factors orthogonal to DM can lead to similar physiological and behavioral effects. Despite these limitations, we believe our work suggests that a useful screening or detection tool is possible and may be further adapted and developed in a clinical study.

## Conclusion

Our work demonstrates that the application of algorithms for detecting people at high risk for DM using longitudinal, noninvasive wearable data is feasible in a large, retrospective population. Noninvasive physiological data that capture behavioral patterns (sleep) in addition to physiological patterns (HR and distal body temperature) longitudinally improve our ability to track transitions into DM-like physiological states. Sensor modalities that are less widely adopted across noninvasive wearable health devices, like skin temperature, can improve detection algorithms with well-selected features. Future work may leverage CGM to incorporate the temporal relationship between hyperglycemic/hypoglycemic events and the apparent physiological states of our identified true positive class. Our work moves the needle towards passive screening for DM risk from a broad array of non-specialized devices, as well as passive, noninvasive monitoring to complement CGM for those in need early in their condition. Our findings suggest that a variety of chronic conditions could be detected from a non-specialized device by repeatedly measuring features relevant to the effects of a particular chronic condition over time.

## Supplementary information

Supplemental Information

Description of Additional Supplementary Files

Supplementary Data 1

Supplementary Data 2

Supplementary Data 3

## Data Availability

Oura’s data use policy does not permit us to make wearable device data (collected via the Oura Ring) available to third parties. Access to anonymized and privacy-protected data may be granted to a qualified academic investigator upon completing agreements with Oura Health Oy and the investigators. Please contact Ashley E. Mason and Benjamin L. Smarr to obtain an application to obtain these data. The data tables used to produce figures are publicly available at the following GitHub page: https://github.com/Zuko09/tempredict_detecting_diabetes_from_wearable_data^58^ with all proprietary data (and figures or tables of this data), personally identifiable information, and personal health information removed.

## Code availability

The underlying code for this study and data tables used to produce figures are publicly available at the following GitHub page: https://github.com/Zuko09/tempredict_detecting_diabetes_from_wearable_data^58^. We have removed proprietary data (and figures or tables of this data), personally identifiable information, and personal health information.
