# Supplementary material for: Sleep and temperature data from wearable devices support noninvasive detection of diabetes mellitus in a large-scale, retrospective analysis
Source: Commun Med (Lond). 2026 Mar 16;6:223. doi: 10.1038/s43856-026-01501-0 (PMC13079903; doi:10.1038/s43856-026-01501-0)
Supplement: Supplementary file 1 — Supplemental Information [file 43856_2026_1501_MOESM1_ESM.pdf]

Supplementary Tables

**Supplementary Table 1: P-values from Posthoc Dunn's Test Comparing Predicted Probabilities of Analysis of Other Chronic Condition Groups**				
**Feature Sets Used**	**P-values**			
**Sets 1-3**		**People Self-Reporting No Chronic Conditions** (n = 38)	**People Self-Reporting no DM DX with Other Chronic Conditions** (n = 18)	**People Self-Reporting DM** (n = 47)
	**People Self-Reporting No Chronic Conditions**	1.0000	0.9823	**8.0e-6**
	**People Self-Reporting no DM DX with Other Chronic Conditions**	0.9823	1.0000	**0.0003**
	**People Self-Reporting DM**	**8.0e-6**	**0.0003**	1.0000
**Sets 1-5**		**People Self-Reporting No Chronic Conditions** (n = 38)	**People Self-Reporting no DM DX with Other Chronic Conditions** (n = 18)	**People Self-Reporting DM** (n = 47)
	**People Self-Reporting No Chronic Conditions**	1.0000	0.3671	**6.9e-12**
	**People Self-**	0.3671	1.0000	**7.0e-6**

	**Reporting no DM DX with Other Chronic Conditions**			
	**People Self-Reporting DM**	**6.9e-12**	**7.0e-6**	1.0000

Supplementary Table 1: P-values from Posthoc Dunn's test comparing predicted probabilities of Analysis of Other Chronic Condition groups from test data of models trained with different input features. Each sample is the average predicted probability for an individual. Comparisons with significant p-values ($p < 0.05$) are bolded and columns are bolded.

**Supplementary Table 2: P-values from Posthoc Dunn's Test Comparing False Negatives, True Negatives, and True Positives**				
**Common Features**	**P-values**			
**hr_average**		**FN**<br>(n = 394)	**TN**<br>(n = 3,093)	**TP**<br>(n = 1,746)
	**FN**	1.0000	0.0547	0.5873
	**TN**	0.0547	1.0000	**6.8e-5**
	**TP**	0.5873	**6.8e-5**	1.0000
**rmssd**		**FN**<br>(n = 394)	**TN**<br>(n = 3,093)	**TP**<br>(n = 1,746)
	**FN**	1.0000	**0.0307**	0.5399
	**TN**	**0.0307**	1.0000	**9.0e-6**
	**TP**	0.5399	**9.0e-6**	1.0000
**total_sleep_time**		**FN**<br>(n = 394)	**TN**<br>(n = 3,093)	**TP**<br>(n = 1,746)
	**FN**	1.0000	0.9718	0.3474
	**TN**	0.9718	1.0000	**0.0037**

	**TP**	0.3474	**0.0037**	1.0000
**diurnal_distal_body_temperature_amplitude**		**FN** (n = 394)	**TN** (n = 3,093)	**TP** (n = 1,746)
	**FN**	1.0000	0.4666	0.4666
	**TN**	0.4666	1.0000	**6.3e-5**
	**TP**	0.4666	**6.3e-5**	1.0000

Supplementary Table 2: P-values from Posthoc Dunn's Test Comparing False Negatives (FN), True Negatives (TN), and True Positives (TP), where each sample is a prediction on a time window. Comparisons with significant p-values ($p < 0.05$) are bolded and columns are bolded.

**Supplemental Table 3: Impact of Each Feature Set on Classifier Performance**						
		**Feature Set 1**	**Feature Set 2**	**Feature Set 3**	**Feature Set 4**	**Feature Set 5**
**Feature Importance**	**Mean Feature Importance**	0.0469	0.0130	0.0170	0.0181	0.0392
**Feature Ablation**	**Mean Increase in AUROC Upon Addition**	0.0729	0.0009	0.0137	0.0191	0.0724

Supplementary Table 3: Feature importance from XGBClassifier model and feature ablation results across different feature sets. AUROC=Area under Receiver Operating Characteristic.

**Supplementary Table 4: Statistics for Feature Values Split by People Self-Reporting DM and People Self-Reporting No DM DX Groups**				
**Feature**	**Measures of central tendency**	**People rep. No DM DX**	**People rep. DM**	**Effect Size (Cohen's d) (Mann-Whitney U P < 0.0001 for all)**
**All**	**Num. Nights**	764,315	24,486	-
**Heart Rate Lowest (BPM)**	**mean**	55.775	61.087	0.657
	**std**	9.281	10.289	
	**mean**	62.713	67.819	0.607

**Heart Rate Average (BPM)**	**std**	8.852	9.569	
**RMSSD (ms)**	**mean**	42.944	30.141	-0.555
	**std**	24.973	17.180	
**REM RMSSD (ms)**	**mean**	39.302	27.032	-0.537
	**std**	24.586	16.641	
**NREM RMSSD (ms)**	**mean**	43.396	30.633	-0.548
	**std**	25.304	17.647	
**Respiratory Rate Average (BrPM)**	**mean**	15.218	15.815	0.396
	**std**	1.583	1.647	
**Distal Body Temperature Average (°C)**	**mean**	35.163	34.779	-0.632
	**std**	0.646	0.842	
**Distal Body Temperature Circadian Amplitude (°C)**	**mean**	7.261	6.008	-0.455
	**std**	2.848	2.438	
**Total Sleep Time (hrs)**	**mean**	7.946	7.649	-0.192
	**std**	1.726	1.990	
**Sleep Efficiency**	**mean**	0.855	0.843	-0.156
	**std**	0.090	0.107	

Supplementary Table 4: Mean and standard deviation for features around heart rate, heart rate variability and sleep efficiency. Statistics were calculated using features extracted at the nightly level. BPM = Beats per minute, ms = milliseconds, BrPM = Breaths per minute, °C = Degrees Celsius, hrs = hours, std = standard deviation, People rep. DM = people who self-reported diabetes mellitus, People rep. no DM DX = people self-reporting no diagnosis of diabetes mellitus.

Supplementary Table 5: Performance metrics on Testing Data for each combination of Classifier and Input Time Window

		**Metrics**							
**Clf.**	**Window (Days)**	**Precision**	**Recall/ Sensitivity**	**Specificity**	**F1 Score**	**TPR**	**FPR**	**AUROC**	**AUPRC**
**XGB Clf.**	**1**	0.609	0.848	0.543	0.709	0.848	0.457	0.741	0.666
	**3**	0.657	0.787	0.751	0.716	0.787	0.249	0.844	0.764
	**7**	0.678	0.798	0.777	0.733	0.798	0.223	0.863	0.774
	**14**	0.689	0.824	0.785	0.751	0.824	0.215	0.881	0.794
	**21**	0.705	0.816	0.809	0.756	0.816	0.191	**0.881**	**0.801**
**Log. Reg.**	**1**	0.592	0.927	0.463	0.723	0.927	0.537	0.733	0.668
	**3**	0.666	0.871	0.735	0.755	0.871	0.265	0.870	0.778

	**7**	0.703	0.838	0.792	0.765	0.838	0.208	0.874	0.776
	**14**	0.703	0.851	0.792	0.770	0.851	0.208	**0.878**	**0.783**
	**21**	0.696	0.851	0.792	0.766	0.851	0.208	0.877	0.776

Supplementary Table 5: Performance metrics on testing data for each classifier. Threshold selected by Youden's J statistic from ROC curve. AUROC=Area under Receiver Operating Characteristic, AUPRC=Area under Precision Recall Curve, TPR=True Positive Rate, FPR=False Positive Rate, Clf=Classifier, Log Reg= Logistic Regression. XGB Classifier with 21-day input time windows was selected as best performing classifier. Strongest performing AUROC and AUPRC are bolded.

**Supplementary Table 6: Performance metrics on Imbalanced Cohort Testing Data for each combination of Classifier and Input Time Window**									
		**Metrics**							
**Clf.**	**Window (Days)**	**Precision**	**Recall/ Sensitivity**	**Specificity**	**F1 Score**	**TPR**	**FPR**	**AUROC**	**AUPRC**
**XGB**	**1**	0.217	0.887	0.303	0.349	0.887	0.697	0.639	0.336
	**3**	0.322	0.772	0.640	0.452	0.772	0.360	0.785	0.457
	**7**	0.293	0.544	0.710	0.379	0.544	0.290	0.666	0.371
	**14**	0.326	0.792	0.640	0.461	0.792	0.360	0.795	**0.471**
	**21**	0.324	0.806	0.631	0.461	0.806	0.369	**0.797**	0.469

Supplementary Table 6: Performance metrics on imbalanced cohort testing data for the best classifier. Values represent average of 100 runs. Threshold selected by Youden's J statistic from ROC curve. AUROC=Area under Receiver Operating Characteristic, AUPRC=Area under Precision Recall Curve, TPR=True Positive Rate, FPR=False Positive Rate, Clf=Classifier, Log Reg= Logistic Regression. Strongest performing AUROC and AUPRC are bolded.

**Supplementary Table 7: Demographic information of Ethnicity with Respect to Age**										
	**Ethnicity**									
**Age Bin**	**African**	**Asian**	**Black**	**Ethnic Other**	**Middle Eastern**	**multiple**	**Native American**	**Native Hawaiian**	**South Asian**	**White**
**30-39**	4	164	31	42	31	70	4	3	41	1524
**40-49**	4	145	55	65	12	57	7	7	55	1997
**50-59**	2	54	43	49	10	37	0	2	12	1635
**60-69**	0	17	13	28	4	10	0	1	3	817
**70-79**	0	2	1	11	1	1	1	0	0	299

Supplementary Table 7: Number of individuals in each demographic and age subset of our dataset.
