# Supplementary material for: Sleep and temperature data from wearable devices support noninvasive detection of diabetes mellitus in a large-scale, retrospective analysis
Source: Commun Med (Lond). 2026 Mar 16;6:223. doi: 10.1038/s43856-026-01501-0 (PMC13079903; doi:10.1038/s43856-026-01501-0)
Supplement: Supplementary file 2 — Description of Additional Supplementary Files [file 43856_2026_1501_MOESM2_ESM.docx]

**Description of Additional Supplementary Files**

Supplementary Data 1: Feature descriptions of feature sets 1, 2, and 4. BPM = Beats per minute, BrPM = Breaths per minute, hrs = hours, s = seconds, ms = milliseconds, °C = Degrees Celsius.

Supplementary Data 2: Feature descriptions of the secondary sleep features that are closely based on features from work by Katori et al. and Viswanath et al. hrs = Hours.

Supplementary Data 3: Feature descriptions for feature set 5, the secondary temperature features consisting of the complexity features and the circadian rhythm features. °C = Degrees Celsius.
